# Is PPARβ/δ a Retinoid Receptor?

**DOI:** 10.1155/2007/73256

**Published:** 2008-01-17

**Authors:** Daniel C. Berry, Noa Noy

**Affiliations:** Department of Pharmacology, Case Western Reserve University School of Medicine, 10900 Euclid Ave., Cleveland, OH 44106, USA

## Abstract

The broad ligand-binding characteristic of PPARβ/δ has long hampered identification of physiologically-meaningful ligands for the receptor. The observations that the activity of PPARβ/δ is supported by fatty acid binding protein 5 (FABP5), which directly delivers ligands from the cytosol to the receptor, suggest that *bona fide* PPARβ/δ ligands both activate the receptor, and trigger the nuclear translocation of FABP5. Using these criteria, it was recently demonstrated that all-*trans*-retinoic acid (RA), the activator of the classical retinoic acid receptor RAR, also serves as a ligand for PPARβ/δ. Partitioning of RA between its two receptors was found to be regulated by FABP5, which delivers it to PPARβ/δ, and cellular RA binding protein II (CRABP-II), which targets it to RAR. Consequently, RA activates PPARβ/δ in cells that display a high FABP5/CRABP-II expression ratio. It remains to be clarified whether compounds other than RA may also serve as endogenous activators for this highly promiscuous protein.

## 1. INTRODUCTION

Peroxisome proliferator-activated receptors (PPARs), the “lipid sensors,” are activated by fatty acids and various fatty acid derivatives to regulate the expression of genes involved in multiple physiological functions. Like other members of subclass 1 of the superfamily of nuclear hormone receptors, PPARs interact with the retinoid X receptor (RXR) to form heterodimers that bind to PPAR response elements (PPRE) in regulatory regions of specific target genes [1]. Binding of cognate ligands to these heterodimers results in receptor activation and in upregulation of target gene transcription [2–5]. Three PPAR subtypes, encoded for by three separate genes, are known to exist: PPAR*α*, PPAR*β*/*δ*, and PPAR*γ* [6]. PPAR*α* is expressed in liver, heart, muscle, and kidney, where it regulates fatty acid catabolism. Consequently, activation of PPAR*α* lowers serum lipid levels; and synthetic ligands for this receptor are efficient therapeutic agents in treatment of hyperlipidemia [7, 8]. PPAR*γ* is expressed predominantly in adipose tissue and macrophages, where it is involved in adipocyte differentiation, regulation of sugar and lipid homeostasis, and control of inflammatory responses [9, 10]. Thiazolidinediones, synthetic compounds that activate PPAR*γ*, are in current use as antidiabetic drugs [11]. Several biological lipids have been suggested to serve as endogenous ligands for PPARs. Thus, it was reported that PPAR*α* can be activated by 8(S)-HETE and by leukotriene B4 (LTB4) [12, 13]. It was also suggested that the arachidonic acid metabolite 15 deoxy-delta 12,14 prostaglandin J2 (PGJ2) functions as an endogenous ligand for PPAR*γ* [14]. However, the involvement of these and other potential ligands in the activities of PPARs in vivo has not been established, and these receptors remain classified as “orphan receptors.”

PPAR*β*/*δ* is ubiquitously expressed with particularly high level of expression found in brain, adipose tissue, skeletal muscle, and skin [15]. This receptor is involved in neuronal development [16], inflammation [17–19], skeletal muscle lipid oxidation [20], keratinocyte differentiation, epidermal barrier recovery, and lipid synthesis for keratinocyte proliferation [21, 22]. PPAR*β*/*δ* expression in skin is increased in hyperproliferative lesions, and in response to inflammatory cytokines during skin injury [18, 19]. Elevation of PPAR*β*/*δ* expression in keratinocytes during skin injury is accompanied by production of an (unknown) endogenous ligand(s), resulting in protection against apoptotic signals and in enhancement of skin repair [18]. These antiapoptotic activities are mediated, at least in part, by the ability of PPAR*β*/*δ* to directly upregulate the expression of PDK1, thereby activating the survival factor Akt1 and protecting keratinocytes from apoptosis induced by cytokines such as TNF*α* [19,21,23,24]. PPAR*β*/*δ* is the primary PPAR isotype in skeletal muscle [25] where it plays a role in fiber formation and maintenance [20] and enhances fatty acid oxidation and mitochondrial respiration [26].

Importantly, it has been reported that PPAR*β*/*δ* is involved in regulating lipid and sugar homeostasis and that activation of the receptor results in protection against adiposity and insulin resistance. These activities appear to stem from transcriptional regulation of various genes, including uncoupling proteins 1 and 3, long-chain and very-long-chain acyl CoA synthetase, and muscle carnitine palmitoyltransferase-1, resulting in depletion of adipose lipid storage and in decreased levels of circulating triglycerides and free fatty acids [26]. In macrophages, activation of PPAR*β*/*δ* induces the expression of ALDH9, a gene involved in carnitine biosynthesis, and leads to lipid mobilization and increased fatty acid catabolism [27]. It was also demonstrated that treatment with a selective PPAR*β*/*δ* ligand leads to improvement in hepatic glucose output, higher glucose disposal, and inhibition of free fatty acid release from adipocytes [26]. Protective activities of PPAR*β*/*δ* against insulin resistance may also be mediated by the direct target gene PDK1, which is involved in mobilization of the insulin-responsive glucose transporter GLUT4 to the plasma membrane [28].

## 2. PROPOSED LIGANDS FOR PPAR*β*/*δ*

Like other PPARs, the nature of endogenous, physiologically-meaningful ligands for PPAR*β*/*δ* has long remained unknown. The ligand-binding pocket of this receptor is considerably larger than other nuclear receptors displaying a total volume of ~1300 Å [29–31]. The pocket forms a “Y” shape comprised of three arms approximately 12 Å in length [30, 31]. A solvent-exposed channel allows accessibility into the ligand binding pocket with an entrance area of approximately 100 Å with the ability to open even larger due to the flexibility in surrounding helices [30]. The extensive size of the pocket is consistent with the promiscuous ligand binding displayed by the receptor; and raises the possibility that it may be activated by multiple compounds. Examination of X-ray crystal structures of bacterially expressed PPAR*β*/*δ* ligand-binding domain revealed that the protein was bound by fatty acids such as eicosapentaenoic acid (EPA) [30], 11,Z-octadecenoic acid [31], palmitic acid, and stearic acid [31]. It is worth noting however that, while multiple fatty acid derivatives can bind to this receptor, not all of these function as activators. Biological compounds that were reported to activate PPAR*β*/*δ* include various leukotrienes and prostaglandins, such as prostaglandin A1, iloprost, PG15d-J2, and carbaprostacyclin [32, 33].

## 3. RETINOIC ACID IS A LIGAND FOR PPAR*β*/*δ*

**Figure 1:**
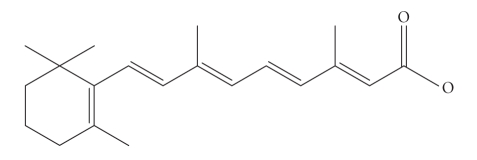
All-trans-retinoic acid.

Recent observations suggested that PPAR*β*/*δ* is activated by an unexpected fatty acid, the vitamin A metabolite all-*trans* -retinoic acid (RA) (see Figure 1). It was thus reported that RA binds to PPAR*β*/*δ* with a Kd of ~15 nM, displaying an order of magnitude higher affinity for this subtype as compared to its affinity towards PPAR*α* and PPAR*γ* [34]. It may be worth noting that the binding affinity for RA towards PPAR*β*/*δ* is over an order of magnitude weaker than the affinity of RAR towards this ligand, which was reported to be in the sub-nM range [35]. Nevertheless, RA enhances the ability of PPAR*β*/*δ*, but not other PPAR isotypes, to induce the expression of a reporter gene driven by a PPRE [34], suggesting that it functions as a selective PPAR*β*/*δ* ligand.

RA plays key roles both during embryonic development and in adult tissue, where it is involved in regulation of cellular metabolism, proliferation, differentiation, and apoptosis. It is well established that many of the pleiotropic activities of RA are exerted primarily through its ability to regulate gene expression, and are mediated by the nuclear hormone receptors termed retinoic acid receptors (RARs) [36, 37]. Like PPARs, RARs heterodimerizes with RXR, the complex binds to RAR response elements (RAREs) in regulatory regions of target genes, and it enhances transcriptional rates upon ligand-induced activation. In this fashion, RA also displays distinct anticarcinogenic activities mediated by RAR-induced upregulation of genes involved in cell cycle arrest [38], differentiation [39, 40], and apoptosis [41–43].

The observations that, in addition to activating RAR, RA also activates PPAR*β*/*δ*, raise the possibility that some of the biological activities of this hormone may be mediated by an RAR-independent pathway. An RAR-independent activity of RA has indeed been suggested by studies of RA functions in skin maintenance. Hence, while it is well established that RA is essential for skin maintenance [44, 45], it was reported that all RAR subtypes are dispensable for this activity [46]. In addition, while activation of RAR often results in inhibition of cell growth [47–49], various reports demonstrated that, in some cancers, this hormone induces carcinoma cell proliferation [50], again suggesting a mode of action that is mediated by a pathway other than activation of RAR. Taken together, the identification of RA as a potent ligand for PPAR*β*/*δ*, and the observations that this receptor can directly upregulate the expression of prosurvival and proproliferative genes [24, 51] raise the intriguing possibility that “nonclassical” proproliferative activities of RA may be mediated by PPAR*β*/*δ*.

Indeed, it was demonstrated that, in keratinocytes, RA functions as a * bona fide* PPAR*β*/*δ*-ligand to induce the expression of well-known PPAR*β*/*δ*-target genes including adipose differentiation-related protein (ADRP) [52], fasting-induced adipose factor (FIAF) [53], and PDK1 [24]. As expression of these genes was found to be induced by RA and by a selective synthetic PPAR*β*/*δ*-ligand, but not by an RAR-selective ligand [54], the data strongly supported the conclusion that these activities of RA in keratinocytes are mediated by PPAR*β*/*δ* and not by the classical RA receptor RAR. An important question raised by these observations relates to the mechanism by which partitioning of RA between the two receptors may be regulated, that is, what underlies the ability of the hormone to activate RAR in some cells but to target PPAR*β*/*δ* in others?

## 4. RA IS DELIVERED TO PPAR*β*/*δ* BY FATTY ACID-BINDING PROTEIN 5

In addition to associating with nuclear receptors, many hydrophobic compounds bind in cells to members of the family of intracellular lipid binding proteins (iLBP). It has been demonstrated that some iLBPs cooperate with some nuclear receptors in mediating the transcriptional activities of their common ligands [51, 55–58]. For example, it was shown that the iLBP termed cellular retinoic acid binding protein II (CRABP-II) serves to directly deliver RA from the cytosol to the nucleus where it binds to RAR to form a complex that mediates direct “channeling” of the hormone from the binding protein to the receptor. It was demonstrated further that, by directly delivering RA to RAR, CRABP-II significantly enhances the transcriptional activity of the receptor, and dramatically sensitizes cells to RA-induced, RAR-mediated biological activities [56, 59].

In addition to CRABP-II, the iLBP family also includes nine isotypes of fatty acid binding proteins. FABPs are more promiscuous than CRABPs and they bind a variety of fatty acids and fatty acid derivatives [51, 60, 61] displaying ligand-binding selectivities reminiscent of those of PPARs. One FABP, termed FABP5 (K-FABP, mal1, eFABP), was found to cooperate with PPAR*β*/*δ* in a manner similar to that found for the cooperation of CRABP-II with RAR. Specifically, it was shown that, upon binding of PPAR*β*/*δ*-activators, FABP5 mobilizes from the cytosol to the nucleus, and that it delivers the ligand to its cognate receptor through direct interactions, thereby significantly augmenting the transcriptional activity of the receptor [51, 54]. Remarkably, while FABP5 binds various ligands with similar affinities, it mobilizes to the nucleus only in response to specific compounds [51, 54]. Hence, PPAR*β*/*δ* activators display two distinct functions: they activate the receptor, and they trigger the nuclear import of the PPAR*β*/*δ*-associated iLBP, FABP5. The observations that RA not only induces the transcriptional activity of PPAR*β*/*δ* but also activates the nuclear translocation of FABP5 [54] raise the level of confidence that this ligand, indeed, functions as a physiologically meaningful activator for the receptor. It should be noted that, in the context of reporter gene assays, RA may activate both RAR and PPAR*β*/*δ* in the absence of their cognate binding proteins if present at high enough concentrations, but that activation at low concentrations is significantly augmenent by the respective binding proteins (see, e.g., [56]). These observations suggest that the binding proteins are necessary for efficient receptor activation under physiological concentrations of their ligands in vivo.

Available information thus indicates that RA alternatively activates two different nuclear receptors: RAR and PPAR*β*/*δ*, and that the partitioning of this hormone between these receptors is regulated by two iLBPs that selectively cooperate with these receptors, CRABP-II, which delivers RA to RAR, and FABP5, which “channels” RA to PPAR*β*/*δ*. Hence, the relative expression levels of the two binding proteins in different cells will determine the spectrum of genes that can be activated in response to RA, and thus the RA-induced cellular responses. Interestingly, it has been demonstrated that, in the context of two cell types, keratinocytes and MCF-7 mammary carcinoma cells, and in tumors that arise in vivo in the mammary carcinoma mouse model MMTV*neu* , alternate activation of the two receptors lead to opposing biological responses: RA-induced activation of the CRABP-II/RAR pathway results in inhibition of cell growth, while activation of FABP5/PPAR*β*/*δ* enhances proliferation and enables cell survival in the face of potent apoptotic agents [54, 59].

## 5. CONCLUSIONS

The involvement of PPAR*β*/*δ* in regulation of keratinocyte proliferation, skeletal muscle metabolism, inflammation, and lipid homeostasis, together with the recent suggestions that this receptor may be a target for therapeutic strategies in treatment of the metabolic syndrome, emphasize the need for identification of ligands that activate this receptor in vivo. The extensive size of its ligand binding pocket and its broad ligand selectivity raise the possibility that PPAR*β*/*δ* may be activated by multiple compounds, and, perhaps, that different ligands serve in this function in different cells and/or under different physiological circumstances. The observations that efficient activation of this receptor requires cooperation with FABP5, which specifically delivers ligands from the cytosol to nuclear PPAR*β*/*δ*, provide a new powerful criterion for identifying physiologically-meaningful ligands. Hence, such ligands will be required not only to activate the receptor, but also to trigger the nuclear translocation of the binding protein. Using these criteria, it was recently demonstrated that RA is a potent endogenous ligand for PPAR*β*/*δ*. These observations suggest that PPAR*β*/*δ* is a retinoid receptor, functioning similarly to RAR and RXR to regulate gene expression in response to a vitamin A metabolite. The question of whether RA is the sole endogenous ligand for this receptor or whether additional physiologically-meaningful ligands exist remains open and awaits further studies.
